# Genetic structure of the mosquito *Aedes aegypti* in local forest and domestic habitats in Gabon and Kenya

**DOI:** 10.1186/s13071-020-04278-w

**Published:** 2020-08-13

**Authors:** Siyang Xia, Luciano V. Cosme, Joel Lutomiah, Rosemary Sang, Marc F. Ngangue, Nil Rahola, Diego Ayala, Jeffrey R. Powell

**Affiliations:** 1 Department of Ecology and Evolutionary Biology, Yale University, New Haven, Connecticut USA; 2 Arbovirus/Viral Hemorrhagic Fever Laboratory, Center for Virus Research, Kenya Medical Research Institute (KEMRI), Nairobi, Kenya; 3 CIRMF, Franceville, Gabon; 4 ANPN, Libreville, Gabon; 5 MIVEGEC, IRD, CNRS, Univ. Montpellier, Montpellier, France

**Keywords:** *Aedes aegypti*, Forest and domestic habitat, Domestication, Africa, Population genetic structure

## Abstract

**Background:**

The mosquito *Aedes aegypti* is a devastating disease vector transmitting several important human arboviral diseases. In its native range in Africa, the mosquito can be found in both the ancestral forest habitat and anthropogenic habitats such as villages. How do the different habitats impact the population genetic structure of the local mosquito populations?

**Methods:**

To address this question, we simultaneously sampled *Ae. aegypti* from the forest and local villages in La Lopé, Gabon and Rabai, Kenya. The mosquitoes were genotyped at 12 microsatellite loci and a panel of ~25,000 single nucleotide polymorphisms (SNPs), which allowed us to estimate their genetic ancestries and the population genetic structure related to habitats and sampling sites.

**Results:**

In the context of the global population genetic structure of *Ae. aegypti*, clustering analysis showed that mosquitoes from the same locality (La Lopé or Rabai) have similar genetic ancestry, regardless of their habitats. Further analysis at the local scale also found no strong genetic differentiation between the forest and village mosquitoes in both La Lopé and Rabai. Interestingly, these results from our 2017 samples from Rabai, Kenya contrast to the documentation of genetic differentiation between village and forest mosquito collections from 1975–1976 and 2009. Between-habitat measures of genetic difference (*F*_*st*_) vary across the genome, with a peak of high divergence observed at the third chromosome only in the La Lopé populations.

**Conclusion:**

Collectively, these results demonstrated that there is little genetic isolation between forest and village habitats, which suggests possible extensive gene flow between them. From an epidemiological perspective, the forest habitat could act as a refuge for mosquitoes against vector control programmes in the domestic settings. Moreover, sylvatic populations could play a role in zoonotic pathogen transferred to humans. Therefore, future studies on disease transmission and vector control planning in the study area should take natural populations into consideration.
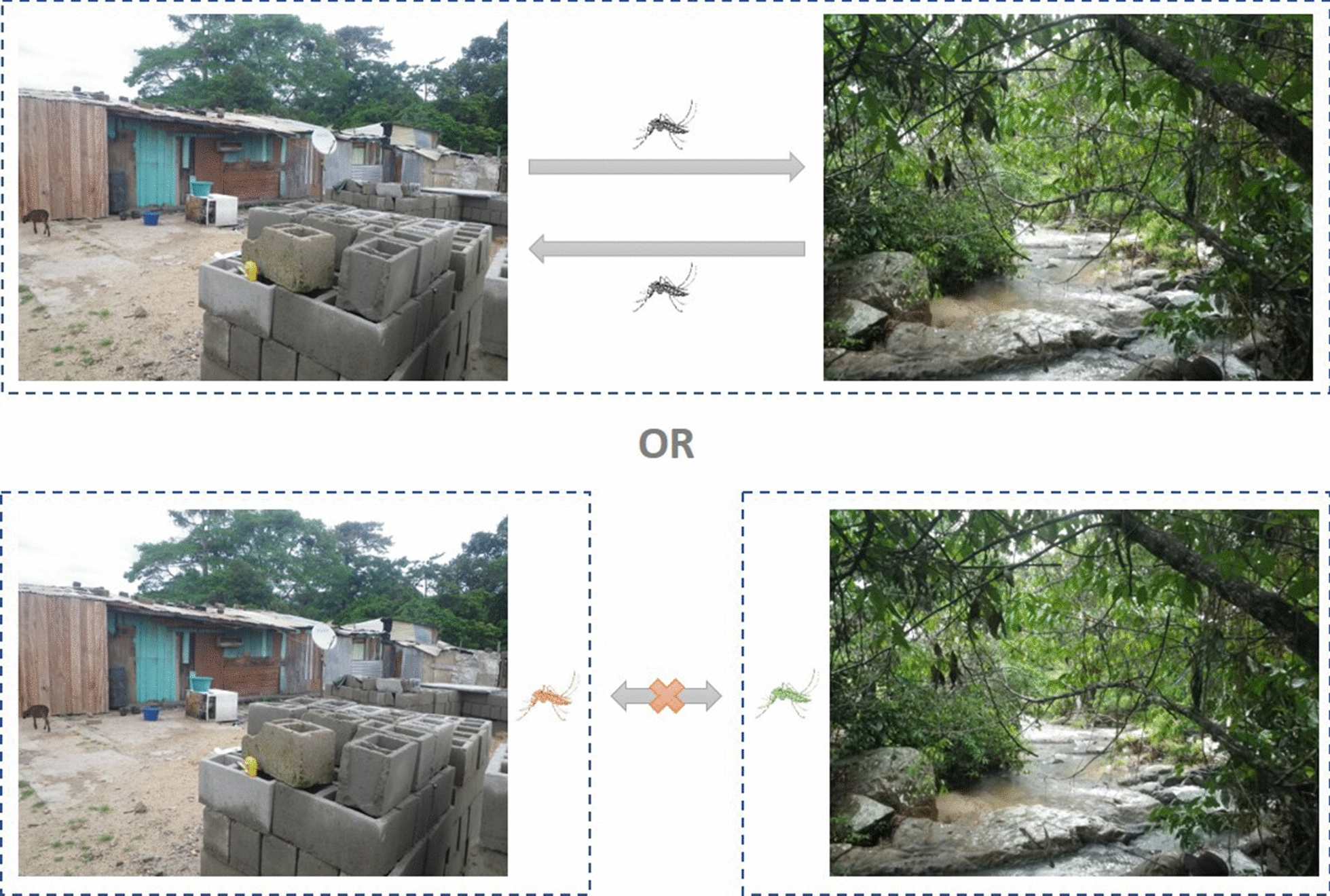

## Background

Anthropogenic habitats such as villages and urban areas have been exploited by many species, including disease vectors [1, 2]. Among them, one of the most successful and notorious examples is the mosquito, *Aedes aegypti*, the main disease vector of several arboviral diseases, including yellow fever, dengue, chikungunya and Zika [3, 4]. Therefore, the mosquito has become a major public health concern in tropical and subtropical regions worldwide [5]. This mosquito is native to sub-African forests and invaded domestic habitats before spreading to other continents in the last 400–500 years, likely associated with human movements such as the slave trade [6–12]. Populations of *Ae. aegypti* outside of Africa exhibit strong adaptations to the domestic environments, for example, a strong preference for biting humans and breeding in human-made containers [13]. These domestic adaptations likely help the mosquitoes to reside permanently around humans, which contributes to their high efficacy as human disease vectors.

Genetic data accumulated during the last few decades consistently suggested strong genetic differentiation between the ancestral populations of *Ae. aegypti* in Africa and the derived populations outside of Africa [7–10, 14]. These two genetic groups roughly match the conventional description of the two subspecies, *Ae. aegypti formosus* (*Aaf*) in Africa with darker body color and *Ae. aegypti aegypti* (*Aaa*) outside Africa with lighter body color [15, 16]. Although subspecies identification is not always clear-cut, here we will use these terms to represent the two major genetic clusters of *Ae. aegypti*. *Aaf* can be frequently found in the forest in Africa, while *Aaa* is specialized in domestic habitats in other continents [9]. However, this match between genetic clusters, geographical locations, and habitats is not perfect: morphologically and genetically defined *Aaf* has been found in many villages and cities (i.e. domestic habitats) in Africa [10, 17–19]. These findings raise an interesting question: what is the impact of habitat shift on the genome of *Ae. aegypti*?

Habitat differences, in theory, could lead to divergent adaptive selection and reduced gene flow allowing independent genetic drift, both contributing to genetic differentiation. While the latter generates genomic-wide differentiation, the former could lead to accelerated rates of divergence in certain parts of the genome that are either directly under selection or tightly linked to the genomic regions under selection [20]. These regions may include genes that mediate the interaction between the animals and their environments, such as genes related to host preference, oviposition, or responses to anthropogenic stressors (e.g. insecticide) in *Ae. aegypti* [21–25]. On the other hand, if the mosquitoes are predisposed to invade different habitats and there is frequent gene flow between habitats, genetic differentiation between habitats will be weak, which could explain the genetic similarities between the forest and domestic *Aaf* populations in Senegal and Cameroon [17–19]. If this is the case, the exploitation of the different habitats is not due to or not necessarily dependent on genetic specialization.

To address this question and to further our understanding of the domestication history of *Ae. aegypti*, we examined the genetic structure of *Ae. aegypti* in La Lopé, Gabon (Central Africa) and Rabai, Kenya (East Africa). Both locations have *Ae. aegypti* living in nearly sympatric forest and village habitats, and the two locations are sufficiently distant from each other that they represent two independent habitat shift events. The mosquito populations in La Lopé were previously sampled in 2014 and genetically classified as *Aaf* [10]. The story in Rabai, Kenya is more complicated. Previous studies have found that mosquitoes living inside residential houses in Rabai are likely descendants of *Aaa* outside of Africa that were introduced to the east coast of Kenya [9, 11, 12]. They lived only indoor and remained genetically distinct from the *Aaf* populations in the nearby forest [26–28]. A third type of *Ae. aegypti* population in Rabai has been proposed as “peridomestic” living in human-disturbed areas such as gardens between the huts and forests, and is similar to what has been observed in village settings at La Lopé. However, the mosquito populations in Rabai, Kenya had not been monitored genetically since 2011 [12]. Re-examining the genetic structure of the forest and village mosquitoes in Rabai could provide updates on this unique case in the evolutionary history of *Ae. aegypti*. More importantly, if this co-existence of the forest, peridomestic, and domestic types prevails, it would present a contrast to La Lopé, Gabon (no evidence of external introduction) to study the genomic evolution associated with habitat difference.

Specifically, the primary goals of this study are to examine: (i) whether forest and village *Ae. aegypti* in each of the two localities shared similar genetic ancestry; (ii) how genetically differentiated mosquitoes are in different habitats; (iii) whether certain regions on the genome show parallel habitat-associated genetic differentiation in the two locations (La Lopé and Rabai). We used 12 microsatellite markers and a high-throughput SNP microarray containing ~25,000 SNP loci, both of which have proven to provide high resolution in detecting the population structure of *Ae. aegypti* globally as well as pinpointing the source of local invasion [9, 10, 29–32]. This study adds to our knowledge of the evolution of mosquitoes living in local forest and domestic habitats inside Africa and provides insights on “domestication” history in *Ae. aegypti*.

## Methods

### Mosquito collections

**Fig. 1:**
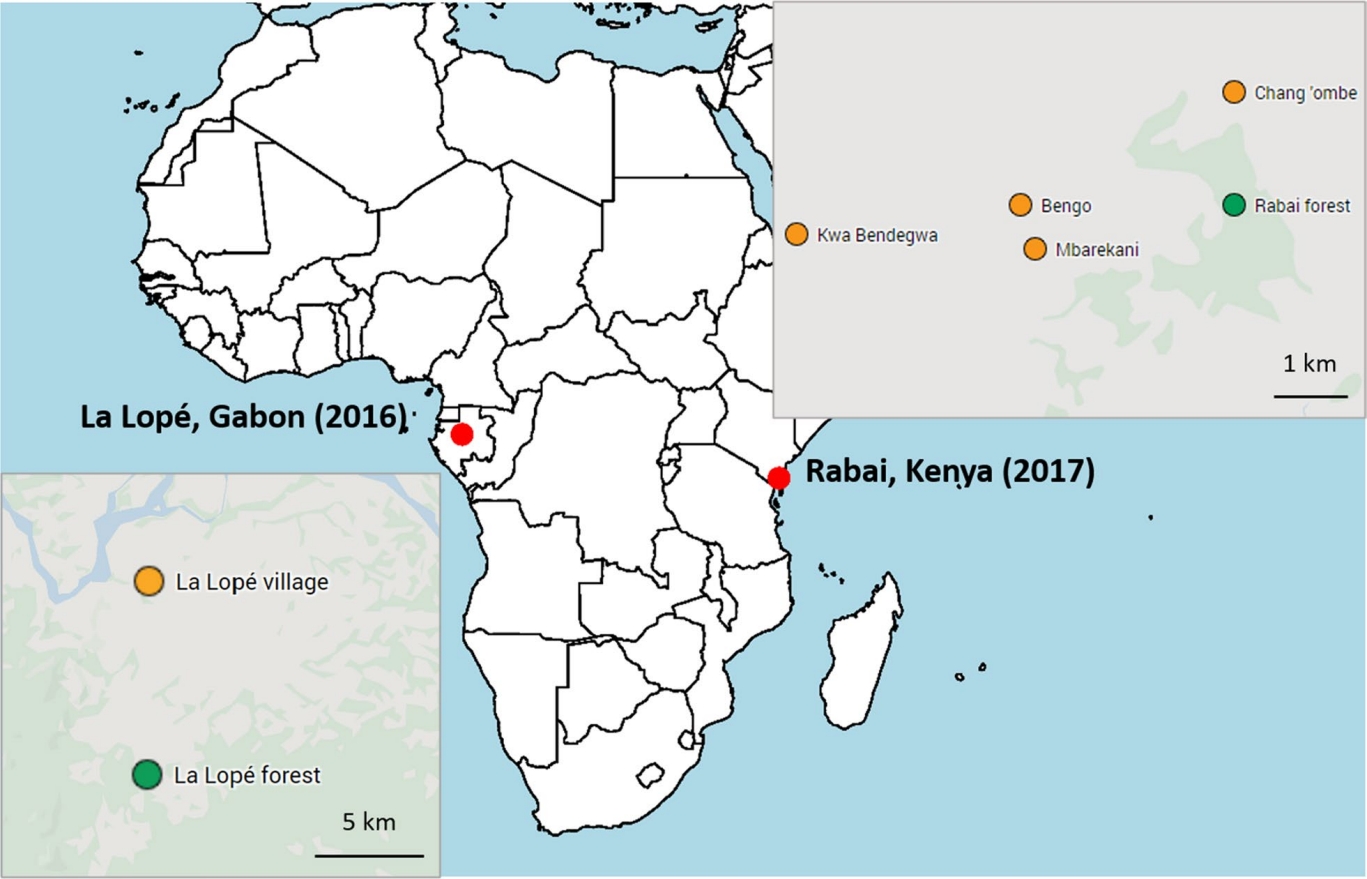
Sampling localities in La Lopé, Gabon and Rabai, Kenya. The green area in the two inset maps roughly represents the forest area. The orange and green dots indicate sampling sites in the villages and the forest, respectively. The continental map was generated in R using *rworldmap*, and the two inset maps are from Google Maps (https://www.google.com/maps)

Mosquitoes were collected from natural breeding sites as larvae in La Lopé, Gabon in November and December 2016 and in Rabai, Kenya in April and May 2017. The sampling sites in La Lopé were at the edge of a continuous tropical rainforest in the La Lopé National Park, where there is a small village (i.e. La Lopé village) about 5–13 km from the forest (Fig. 1). We collected forest *Ae. aegypti* larvae from five rock pools near small streams and two bamboo traps. The seven containers were less than 7 km away from each other. Because of the low abundance of *Ae. aegypti* larvae found in the forest, we supplemented the larvae collection with human landing catches in the forest. We also collected *Ae. aegypti* in La Lopé village from 11 artificial containers like tires and construction bricks scattered no more than 2 km away from each other. In Rabai, Kenya, *Ae. aegypti* were found in tree holes in the forest and artificial containers in four villages (Kwa Bendegwa, Bengo, Mbarekani, and Chang’ombe) about 3–7 km away from the forest (Fig. 1). The 16 tree holes that produced *Ae. aegypti* were within a 1.5 km by 0.6 km forest patch. Within each village, the mosquito breeding sites (3–9 containers per village) were no more than 1 km apart. Because of the relatively low number of breeding sites produced *Ae. aegypti*, we collected all available samples and later used genomic methods to identify and remove siblings (described in the following sections).

In both La Lopé and Rabai, the mosquitoes were reared to adults in the field for species identification. We kept *Ae. aegypti* specimens individually in 80% ethanol at − 20 °C until shipped back to our laboratory at Yale University. Each sample was labeled as one of the three habitat types according to the location of the collection sites: forest, peridomestic (outdoor in the village), and domestic (indoor in the village). In Rabai, we also recorded the geographical coordinate of each individual, which is not available in La Lopé.

### DNA extraction and genotyping

We extracted whole genomic DNA from mosquito samples using the Qiagen Blood and Tissue kit (Qiagen, Hilden, Germany) following the manufacturer instructions. All individuals were amplified and genotyped at 12 microsatellite loci, according to Brown et al. [7]. A subset of samples was submitted to the Functional Genomics Core at the University of North Carolina, Chapel Hill, where they were genotyped using an SNP microarray from Affymetrix designed specifically for *Ae. aegypti* that contains 50,000 loci [29]. Data were analyzed in the Axiom Analysis Suite v.3.1. (Affymetrix, Santa Clara, CA, USA) to generate the genotype calling. We performed genotype calling separately for each analysis that includes different sets of samples as best practice. The SNP data were then filtered in PLINK v 1.9 [33, 34] to remove: (i) loci that have more than 10% missingness; (ii) samples that have more than 10% missing loci; (iii) loci that fail Hardy-Weinberg tests with a threshold of 0.00001 in any population [32]; (iv) loci within a genomic window of 75 kb (moving across the genome) that have a variance inflation factor larger than two; (v) loci that have a minor allele frequency smaller than 1% which could result from genotyping error; and (vi) samples whose expected heterozygosity values deviate more than ± 3 standard deviations from the mean of all samples, which may indicate low quality DNA or high inbreeding [35].

Female *Ae. aegypti* could lay multiple eggs in one container. Therefore, individuals sampled from the same container could be siblings. To identify them, after the filtering, we calculated individual pairwise kinship coefficient *k* as described in Loiselle et al. [36] using the SNP data in SPAGeDi [37]. Siblings were determined when *k* > 0.1875, as in Schmidt et al. [38]. Only one individual per sibling group was retained in the dataset. We then filtered the SNP data again using the same criteria described above in PLINK to generate the final dataset for analysis. We removed the same individuals in our microsatellite dataset as in the SNP data. We also ran SPAGeDi using the microsatellite dataset, which contains more individuals. The analysis resulted in many negative kinship coefficients when individuals were paired with themselves, which suggested that the results of the analysis may not be reliable. Therefore, we did not remove any additional individuals from the microsatellite dataset.

The final number of samples in the microsatellite and SNP dataset are summarized by sampling sites and habitats in Table 1. Sample sizes were constrained by the mosquito density in the field as well as the cost for genotyping. In Rabai, the relatively small sample sizes after partitioning all samples by both sampling sites and habitats restricted their use for many analyses. Therefore, in the following analyses, Rabai samples were grouped by either sampling sites or habitats. For example, when we analyzed the Rabai sample by habitats, we combined the peridomestic samples from all four villages, and domestic samples from all villages, respectively (the second row in Table 1). Alternatively, when we analyze data by locations, we combined the peridomestic and domestic samples within each village (last column in Table 1). The pooling of samples by habitats was inspired by previous studies in the Rabai area showing the mosquitoes indoor and outdoor are genetically distinct [11, 39]. It is possible that geographical separation between villages (Fig. 1) could lead to some hidden genetic differences between villages, which may confound this pooling by habitat. To test this possibility, we thus performed alternative pooling by locations. The results indicate that there is little apparent population structure by location (see below). Because analysis based on a large number of genomic loci is generally less affected by relatively small sample size [40], in this study, we focused more on the SNP-based analysis and used microsatellite data to provide further validation of our findings.

**Table 1:** Sampling sites, habitats, and the number of samples in the microsatellite and SNP dataset

Sampling sites	Forest	Peridomestic (village)	Domestic (village)	Total
La Lopé	47 (11)^a^	36 (8)		83 (19)
Rabai total	60 (11)^c^	22 (12)^c^	40 (18)^c^	122 (41)
Kaya Bomu forest^b^	60 (11)			60 (11)^d^
Chang’ombe^b^		13 (6)	15 (3)	28 (9)^d^
Mbarekani^b^		5 (3)	7 (4)	12 (7)^d^
Bengo^b^		2 (1)	11 (7)	13 (8)^d^
Kwa Bendegwa^b^		2 (2)	7 (4)	9 (6)^d^

^a^Number of samples in the microsatellite data (number of samples in the SNP data)

^b^The forest and the four villages in Rabai, Kenya

^c^Number of samples when grouping Rabai samples by habitats

^d^Number of samples when grouping Rabai samples by sampling sites

Finally, for simplicity, in the rest of the manuscript, we use “populations” to merely refer to groups of mosquitoes collected from the same habitat or sampling site without any prior implication of their genetic identities.

### Inferring genetic ancestry

To first examine the genetic ancestry of the forest and village *Ae. aegypti* in both localities, we clustered our microsatellite and SNP data from La Lopé and Rabai with a representative global genetic dataset of *Ae. aegypti* as references. The reference global microsatellite dataset contains 32 populations, reported in Gloria-Soria et al. [9]. The SNP dataset contains 28 populations worldwide used in Kotsakiozi et al. [10], with the addition of the “Rabai-in, Kenya” population, which is the same as the “Rabai-in, KE” in the microsatellite panel (Table 1 in [9]). This population was collected inside huts (i.e. domestic) in Rabai in 2009 and 2011 and contained individuals classified as *Aaa* genetically. We also updated the name of the population “Kisumu, Kenya” which was originally mislabeled as “Kahawa Sukari” in [10], in order to be consistent with the microsatellite data. Detailed information of all populations is summarized in Additional file 1: Table S1, and their geographical locations are shown in Additional file 1: Figure S1.

For the microsatellite data, we performed a Bayesian clustering analysis using STRUCTURE v 2.3, which generated the probability of each individual attributing to each genetic cluster [41]. We randomly subsampled 30 individuals from each population when there are at least 30 samples available (23 out of 37 populations). For the rest of the populations, all but one have at least 16 individuals. We tested a different number of clusters (*K*) ranging from 1 to 10 and conducted 20 replicates for each *K* with 500,000 MCMC iterations and 100,000 burn-in. The optimal number of clusters was determined by the delta *K* method [42] in STRUCTURE HARVESTER v 0.6.94 [43]. The 20 replicates of each K were summarized and visualized as bar plots using CLUMPP [44] in *pophelper v 2.2.8.1* [45] in R v 3.5.0 [46]. In addition to the STRUCTURE analysis, we performed PCA using the same microsatellite data in the R package *adegenet v 2.1.1* [47, 48].

The SNP data of all populations were filtered the same way as described in the last section. We performed the “snmf” function (sparse non-negative matrix factorization) in the R package *LEA* to estimate the individual ancestry coefficients [49], which produced similar results as STRUCTURE. Five to twelve individuals per population were included except the Rabai domestic 2017 population, which contained 18 samples. The full SNP dataset after filtering contains 23,767 loci. We performed 20 replicates for each *K* from 1 to 34. The best *K* was determined by having the lowest cross-entropy. Finally, the SNP data was also analyzed by PCA in *adegenet*.

### Population structure of La Lopé and Rabai mosquitoes

We next focused on only mosquitoes from the La Lopé 2016 samples or Rabai 2017 samples, which are the main target populations of this study. We first examined how differentiated the mosquitoes living in different habitats or different sampling sites are from each other. The SNP datasets for La Lopé and Rabai consist of 17,694 and 23,068 loci after filtering and removing siblings. Pairwise genetic distance (*F*_*st*_) and genetic differentiation were evaluated in GENEPOP v 4.7 [50, 51] using either the microsatellite or the SNP dataset. Expected heterozygosity (*He*) and observed heterozygosity (*Ho*) were calculated in *adegenet* (microsatellite) and SPAGeDi (SNP). Using the microsatellite data only, we also calculated allelic richness and private allelic richness using HPRARE v1.1 [52]. For Rabai samples, we performed all analyses by pooling habitats and pooling sampling sites, respectively (Table 1). Even after pooling peridomestic and domestic individuals, the sample sizes of three Rabai villages are too small for using microsatellite data to estimate many metrics reliably. Therefore, when we grouped Rabai samples by locations, we only reported the descriptive metrics calculated from the SNP data (Additional file 1: Tables S2, S3).

In addition to these descriptive metrics, we estimated the genetic ancestry of the mosquitoes using STRUCTURE (microsatellite) and LEA (SNP), as described above. We also applied principal components analysis (PCA) in *adegenet* (microsatellite) and LEA (SNP) to summarize the genetic variability in our samples and visualize the genetic distances between individuals.

To further increase the power to detect any genetic structure in our fine spatial scale (3–13 km) within La Lopé or Rabai, we supplemented our population-level analysis with individual-based approaches. Specifically, we compared the kinship coefficient *k* calculated by SPAGeDi (see above) across different categories of individual pairs: within forest (both individuals were collected in the forest), forest-village (one individual came from the forest and the other from the village), within village (both individuals were from the same village), and between villages (the two individuals were from different villages). The last category only applies to the Rabai samples, and both peridomestic and domestic habitats were considered as the village. We did not further distinguish mosquito pairs from the same breeding sites or different breeding sites, because there are very few pairs from the same breeding sites. In addition to the categories by locations, for analysis of the Rabai samples, we also applied an alternative method to categorize the individual pairs by habitats, which resulted in six categories (forest-forest, forest-peridomestic, forest-domestic, peridomestic-peridomestic, peridomestic-domestic, domestic-domestic). Differences between categories were tested by analysis of variance (ANOVA) with *post-hoc* multiple comparisons by t-test and Holm correction for the *P-*values. We used bootstrap with 1000 iterations to estimate the *P*-value of each test to account for the non-independence of the pairwise data.

The relatedness measures could be affected by geographical distances as individuals collected further apart are more likely to be less related. Information about the exact sampling location of each mosquito was available in Rabai but not La Lopé. Therefore, for the Rabai dataset, we examined this potential distance effect using a Mantel test in *adegenet* between the individual pairwise kinship coefficients (estimated with SNPs) and individual pairwise geographical distance, with 999 permutations. To remove this possible distance effect in the Rabai data, we applied a linear regression model. The model used pairwise kinship coefficients as the dependent variable and the pairwise geographical distance as the predictor. We then extracted the residuals from the model and used it to compare between categories described above. By using the residuals, we controlled for possible distance effects on kinship. We did not apply this linear model on the La Lopé dataset as, unfortunately, the geographical information was not available. Instead, we used the raw values of kinship estimates for the La Lopé analysis. If habitat does predict genetic clustering, we would expect reduced gene flow and hence lower genetic relatedness between the forest- and village-living mosquitoes.

Lastly, we constructed a phylogeny that consisted of both La Lopé and Rabai samples with *Aedes mascarensis* as the outgroup, using 22,287 SNPs in IQTree v 1.6.12 [53]. IQTree conducts automatic model selection (option “MFP”) [54], ascertainment bias adjustment (option “ASC”) [55], and model violation check (option “bnni”). We performed ultrafast bootstraps (*n* = 1000) to estimate node certainty [56]. Nodes with support values smaller than 80 were collapsed using R package *ape v 3.5.3* [57, 58].

### Detecting heterogeneous differentiation across the genome

Habitat-associated genetic differentiation, if it exists, could vary across the genome. To examine this possibility, we took advantage of the genome-wide distribution of the SNP loci and calculated *F*_*st*_ between forest and village *Ae. aegypti* on each small segment of the genome (i.e. a genomic “window”). We then slid this “window” through the whole genome, i.e. a genome scan. For simplicity, peridomestic and domestic samples were grouped as village samples. We implemented this sliding-window analysis in VCFtools v 0.1.14 [59] with a window size of 1,000,000 bp and a sliding step of 10,000 bp (i.e. moving the window for 10,000 bp between two consecutive tests). The mean number of SNPs per window is 12.3 in the La Lopé dataset and 16.0 in the Rabai dataset. Windows with fewer than three SNPs were removed, which represented 17.3% and 15.9% of all windows in La Lopé and Rabai populations, respectively.

## Results

### Inferring genetic ancestry

**Fig. 2:**
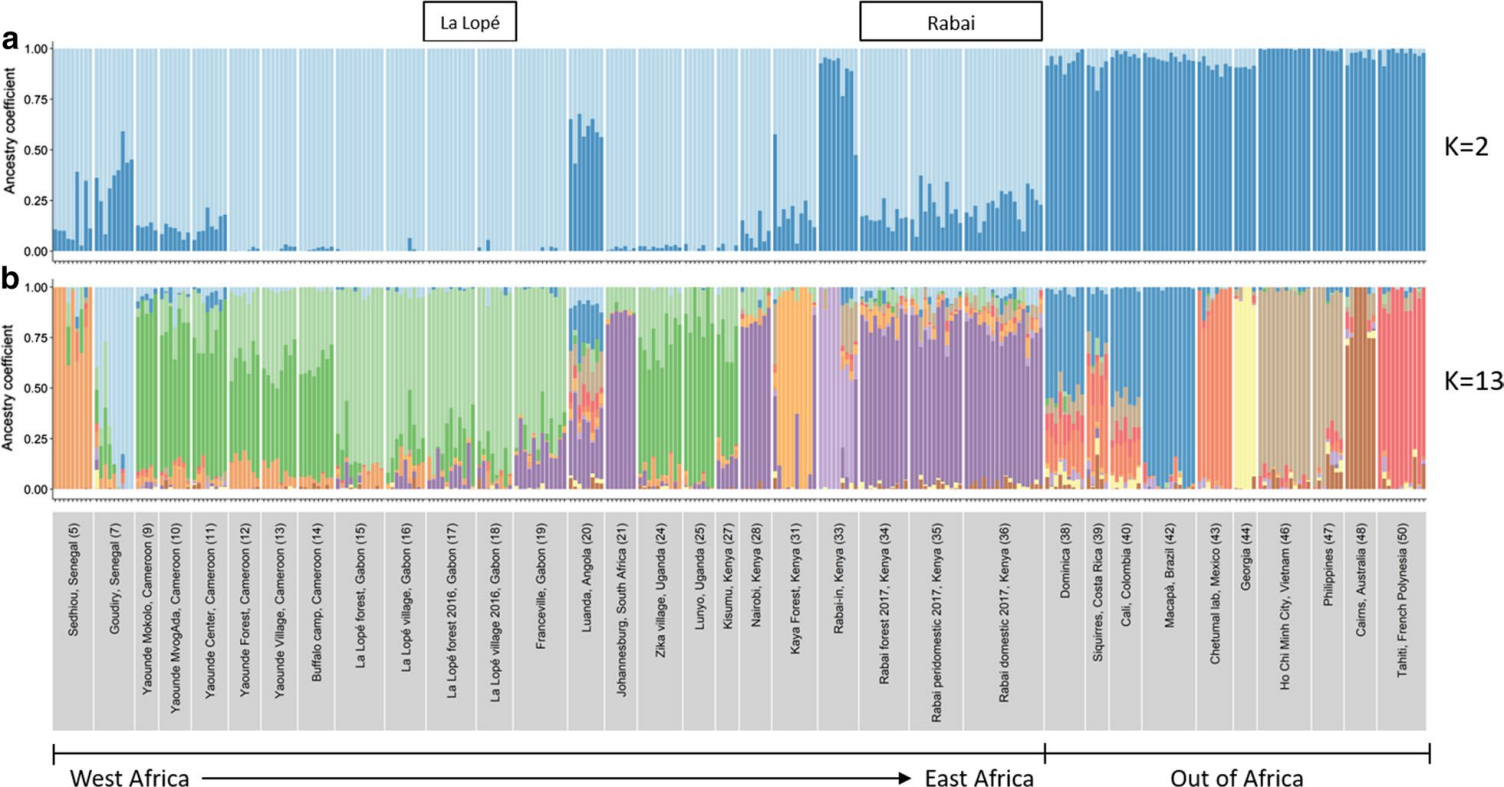
Genetic ancestry of the La Lopé and Rabai populations reported here (boxed names above figure) with a reference panel of the global populations of *Ae. aegypti*, estimated using 23,767 SNP loci in LEA with *K* = 2 (**a**) and *K* = 13 (**b**). Each bar represents one individual. Different colors represent different genetic clusters, and the proportion of a color indicates the probability of an individual assigned to that genetic cluster. Population names and regions are listed on the x-axis. The numbers in the parentheses correspond to the population index in Additional file 1: Table S1 (column “ID”)

LEA and STRUCTURE analysis confirmed that the 2016–2017 collections of *Ae. aegypti* in La Lopé and Rabai were genetically similar to most other African populations. The best number of clusters was 13 for the LEA analysis using SNP data and two for the STRUCTURE analysis using microsatellite data. However, in both analyses, the first split of all samples (i.e. allowing only two clusters or *K* = 2) was between most African populations, including our La Lopé and Rabai samples, and populations outside of Africa (Fig. 2a; Additional file 1: Figure S2a), with only a few exceptions (discussed in detail in Gloria-Soria et al. [9] and Kotsakiozi et al. [10]). This split roughly represents the strong genetic differentiation between *Aaf* and *Aaa*, as suggested in previous studies [7, 9, 11].

The snmf analysis in LEA with *K* = 13 (the best number of clusters, Fig. 2b) demonstrated a more detailed population structure within Africa. La Lopé populations and Rabai populations were significantly genetically distinct, consistent with the hypothesis that they represent independent habitat shift events. The forest and village mosquitoes in La Lopé from 2016 showed very similar genetic ancestries. They also resembled the samples collected from the same location in 2014 (Fig. 2b). As for Rabai samples, all mosquitoes from the 2017 collection clustered together, along with the populations from Nairobi, Kenya, and Johannesburg, South Africa (Fig. 2b). Clustering analysis showed little difference between individuals from different habitats (i.e. forest, peridomestic and domestic). Only minimal traces of the genetic ancestry differing from the main *Aaf* cluster existed in the 2017 Rabai samples, which is the ancestry similar to the Rabai indoor samples from 2009 and 2011 and some Asian-Pacific populations (i.e. an *Aaa* signal). These findings based on the SNP data were consistent with STRUCTURE analysis with microsatellite data (Additional file 1: Figure S2, *K* = 5 to show more genetic clusters within Africa, despite *K* = 2 being the best model). In addition, PCA with microsatellite data and SNP data both showed that the 2016 La Lopé samples and the 2017 Rabai samples overlap with the majority of African populations (Additional file 1: Figure S3), which is again consistent with snmf and STRUCTURE analysis results.

### Population genetic structure of La Lopé and Rabai populations

**Fig. 3:**
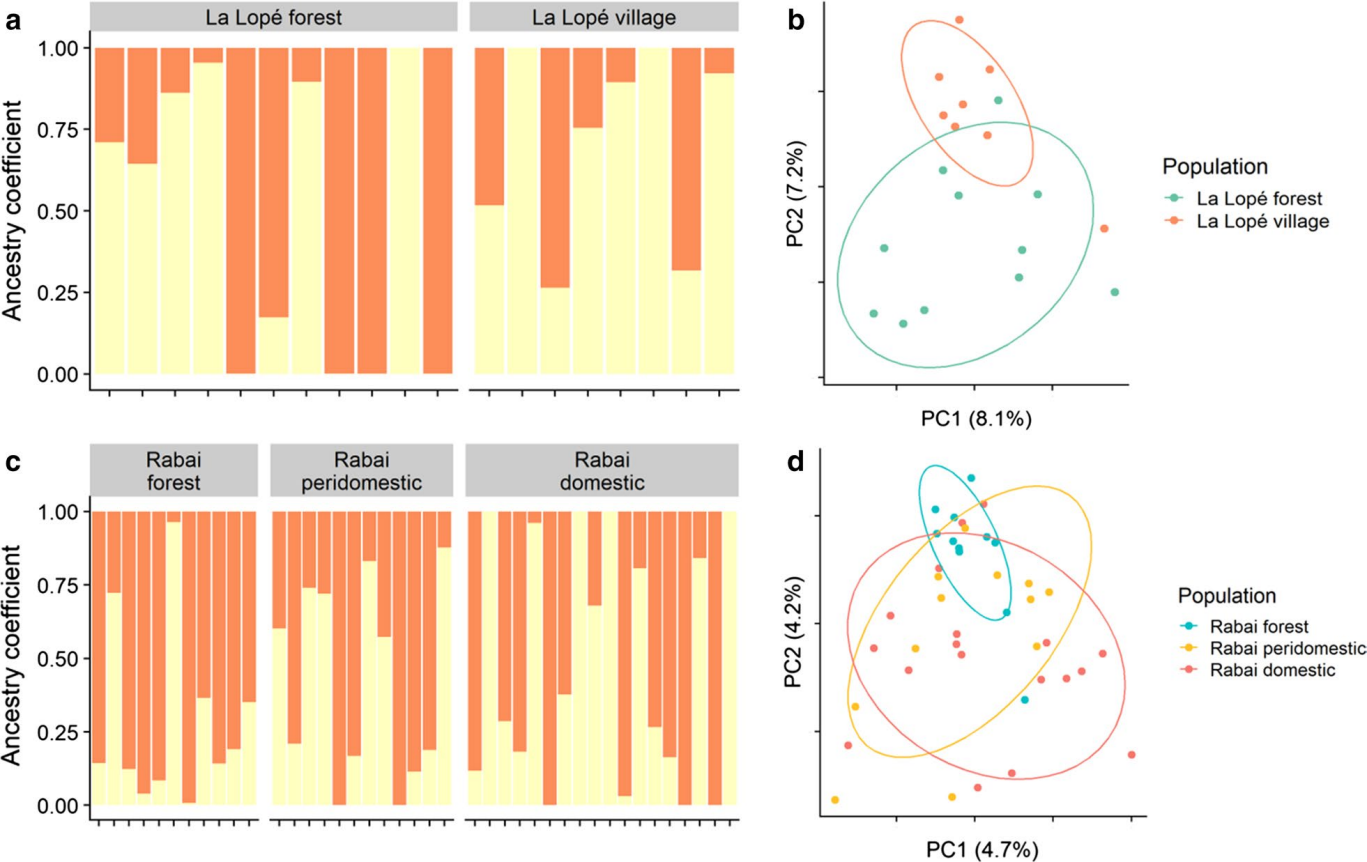
Genetic structure of the La Lopé (**a** and **b**) and Rabai (**c** and **d**) mosquitoes grouped by habitats, generated using 17,694 SNP loci and 23,068 SNP loci, respectively. **a**, **c** Results of the LEA analysis with *K* = 2. The habitats are labeled on top of each bar plot. **b**, **d** PCA biplots by *LEA* showing the first two principal components (PCs). The numbers in parentheses on the axes indicate the percentage of total variation explained by the PCs. The eclipses were drawn with an 80% confidence level

Focusing separately on the La Lopé 2016 and Rabai 2017 populations, the expected and observed genetic heterozygosity (*He* and *Ho*) of mosquitoes from different habitats or sampling sites were comparable (Additional file 1: Table S2). The average allelic richness of the 12 microsatellite loci was also similar between habitats in La Lopé and Rabai, respectively. Private allelic richness also did not differ largely between habitats in Rabai, yet in La Lopé, the forest population had more private alleles than the village population. The pairwise genetic differences (*F*_*st*_) ranged from 0.0066 to 0.0336 when using microsatellite data and from -0.003 to 0.020 when using the SNP data (Additional file 1: Table S3). LEA and STRUCTURE analysis consistently suggested *K* = 1 as the best model for both La Lopé and Rabai populations. When examining results with a larger *K* (Fig. 3; Additional file 1: Figures S4, S5, *K* = 2 as an example), both SNP-based and microsatellite-based analysis did not find evidence of genetic differences between individuals from different habitats or sampling sites, which was also supported by the PCA (Fig. 3; Additional file 1: Figures S4, S5).

**Fig. 4:**
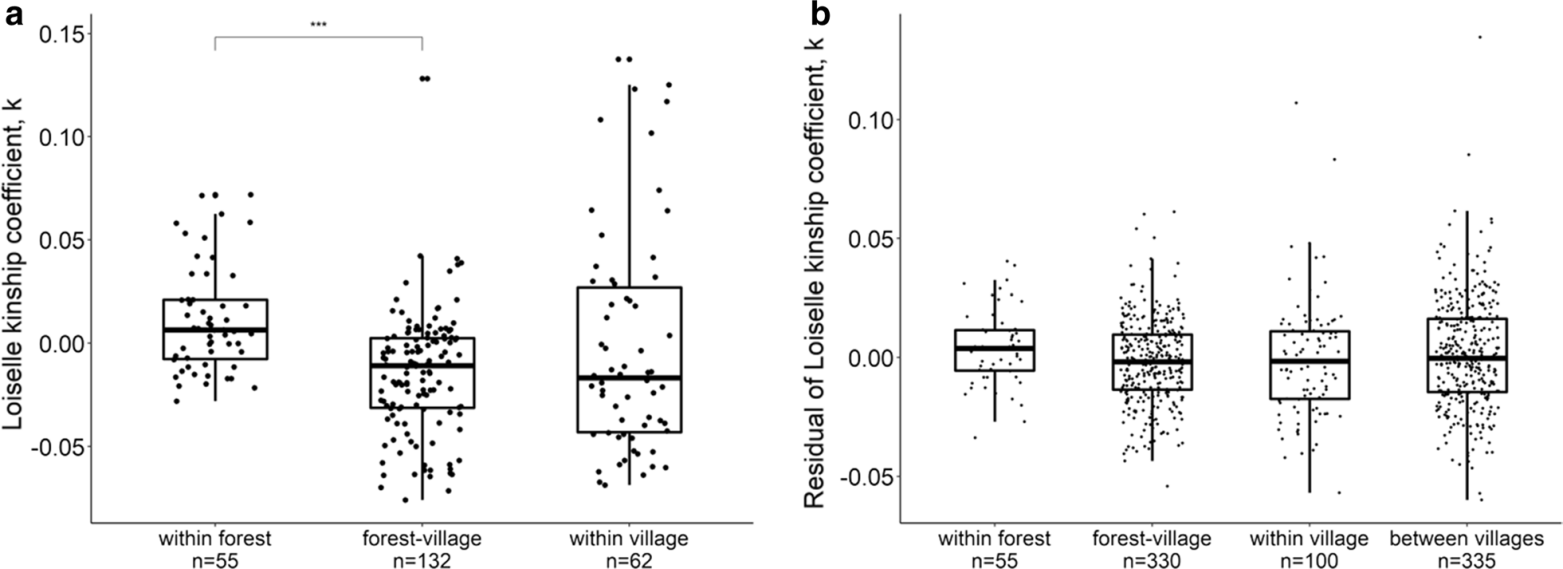
Individual pairwise kinship coefficients of mosquito samples from La Lopé (**a**) and Rabai (**b**). Each point represents one mosquito pair. **a** For La Lopé samples, mosquito pairs are grouped into three categories with the number of pairs labeled on the x-axis. Statistically significant differences between groups are determined by ANOVA with multiple comparisons by t-tests, and indicated by the brackets (significance levels: *P < 0.05, **P < 0.01, ***P < 0.001). **b** Residuals of the kinship coefficients in Rabai after removing distance effects grouped into four categories, with the number of pairs labeled on the x-axis. The boxplots show the median (the horizontal bar), interquartile range (IQR, the box), and 1.5 × IQR above and below the IQR (the vertical bar)

In La Lopé, pairwise kinship coefficients were higher in the “within forest” category than the “forest-village” category (Fig. 4a, ANOVA for all three categories of individual pairs: *F* = 10.31, bootstrap *P* < 0.001, *post hoc* comparison-test: bootstrap *P* < 0.001). Comparisons between these two categories and the “within village” did not show a statistically significant difference (Fig. 4a). Due to the absence of geographical information associated with each mosquito individual, we could not examine the potential effect of geographical distance in La Lopé. Such data were available for the Rabai samples. We found a negative correlation between geographical distance and kinship coefficients (*r* = −0.172, Mantel test *P* = 0.001, Additional file 1: Figure S6a). After removing the effect of distance, ANOVA did not show a significant difference between categories by locations (Fig. 4b, *F* = 1.992, bootstrap *P* = 0.097) or categories by habitats (Additional file 1: Figure S6b, *F* = 1.337, bootstrap *P* = 0.211). None of the pairwise comparisons between categories were significantly different.

The phylogeny constructed from the SNP data shows that mosquitoes from La Lopé and Rabai formed two monophyletic clades, and the split between them was highly supported (Additional file 1: Figure S7). Within the La Lopé clade, village individuals were nested within the forest individuals and form three groups. There was very little well-supported structure in the Rabai clade and no clear pattern of clustering by either habitats or sampling sites (Additional file 1: Figure S7).

### Detecting heterogeneous differentiation across the genome

**Fig. 5:**
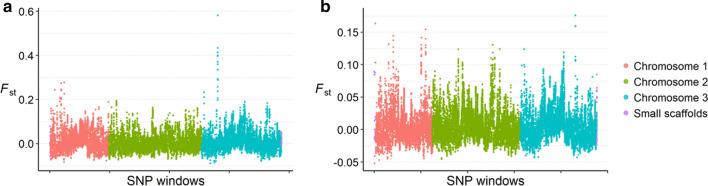
Genetic difference (*F*_*st*_) across the genome between the forest and village mosquitoes from La Lopé (**a**) and Rabai (**b**). Each point represents a 1 Mb window, and the colors indicate the chromosomes

The sliding-window analysis revealed a peak of *F*_*st*_ between the La Lopé forest and La Lopé village populations on chromosome three, centered roughly at nucleotide position 85,800,000 and spanning from position 84,990,000 to 86,960,000 (Fig. 5a). The region was annotated with 16 genes on VectorBase [60] (Additional file 1: Table S4). This region does not stand out in the same analysis for Rabai forest *versus* village samples (Fig. 5b).

## Discussion

The initial results of this study first show that mosquitoes in La Lopé, Gabon and Rabai, Kenya have genetic ancestry more similar to most other African *Ae. aegypti* populations than populations outside of Africa. Within this general African ancestry group (*Aaf*), clustering analysis and the phylogenetic tree suggest a clear distinction between these two locations (Fig. 2; Additional file 1: Figures S2, S7). This finding suggests that the La Lopé and Rabai populations likely evolved independently when colonizing different habitats (forest *vs* villages). This is consistent with the conclusion of Brown et al. [7] suggesting multiple independent domestication events in Africa.

Considering each location separately, there is no strong genetic differentiation between mosquitoes in the forest and village habitats in La Lopé or Rabai. Although tests based on microsatellite loci resulted in statistically significant differences in a few pairs of populations (Additional file 1: Table S3), the *F*_*st*_ values of these pairs are small when compared with the genetic variation of *Ae. aegypti* across Africa [9, 10]. Tests using the SNP data also found no significant difference between populations in different habitats (Additional file 1: Table S3). *F*_*st*_ estimates could be affected by our pooling of samples across the four villages in Rabai that are geographically separated (Fig. 1, Table 1). However, *F*_*st*_ between villages are generally small and not statistically significant (Additional file 1: Table S3), and heterozygosity measures are comparable across different habitat groups (Additional file 1: Table S2). This evidence, combined with the lack of population structure in the clustering analysis, PCA analysis, and phylogenetic analysis (Additional file 1: Figures S4–S7), suggested that pooling samples did not strongly confound our analysis or conclusions. Another potential suggestion of possible genetic differentiation is the higher relatedness between forest individuals in La Lopé (Fig. 4a). However, this may be confounded by the effects of geographic distance. Unfortunately, we do not have the mosquitoes’ geographical coordinates in La Lopé to test this hypothesis. The individual pairs within the La Lopé village have similar levels of relatedness as the across-habitat pairs, which does not support the hypothesis that a barrier of gene flow exists between the forest and village mosquitoes. Collectively, we conclude that there is little solid evidence supporting habitat-associated genetic divergence in either La Lopé or Rabai.

This genetic similarity between habitats may suggest frequent gene flow. This hypothesis is supported by the finding that between-habitat individual pairs have similar levels of relatedness compared to within-habitat pairs, except for one comparison (Fig. 4, see discussion above for this exception). An alternative but not mutually exclusive hypothesis is that the habitat shift (i.e. invasion into a new habitat) happened relatively recently, so there has not been enough time to accumulate detectable genetic differentiation. It is also possible that habitat shifts and/or expansion happen periodically. For example, forest mosquitoes may invade villages seasonally during the dry season seeking standing waters stored in households to lay eggs [11]. It is likely that village mosquitoes in La Lopé and Rabai originated from the nearby forest habitats, as the forest is the ancestral habitat for the species [11, 12]. Yet, the data from the present study cannot rule out the possibility that village mosquitoes move to the forest. Regardless of the direction of migration, the lack of habitat-associated local genetic structure suggests that moving into different environments does not necessarily require, or result in, immediate genetic evolution.

*Aedes aegypti* were collected in both localities before our 2016 and 2017 collections. The 2014 and 2016 samples in La Lopé clustered together in LEA and STRUCTURE analysis (Fig. 2; Additional file 1: Figure S2), which suggests temporal stability. However, in Rabai, the previously documented genetically distinct indoor population (collected in 2009 and 2012) was not detected in 2017 (Fig. 2; Additional file 1: Figure S2). All Rabai 2017 samples showed strong genetic similarity to the “Rabai-out” population in Brown et al. [7] and Gloria-Soria et al. [9] (Additional file 1: Figure S2), which contains forest and peridomestic samples from 2009 and 2012. This result suggests that mosquitoes in the forest and peridomestic habitats remain mostly stable while the indoor domestic mosquitoes were likely assimilated or replaced by the former. A significant recent change in the Rabai villages is that villagers now have access to a centralized covered water source preventing mosquito breeding. This has eliminated the need to store water in open clay pots inside each hut, which were the source of indoor samples in the earlier studies [13, 26, 27, 61]. This likely caused the disappearance of *Aaa* in Rabai. However, it is important to note that the 2017 samples were taken over a single two-week period; it is conceivable that *Aaa* in Rabai varies seasonally and/or still exists in small pockets we did not sample. In addition, we cannot rule out the possibility that the temporal changes in Rabai were due to invasion from external sources. An ongoing project is focusing on better understanding the causes of these temporal variations of Rabai populations and identifying possible sources of external invasions. The findings will be reported in a future manuscript.

The genomic scan revealed variation in *F*_*st*_ between the forest and village mosquitoes along the genome, including a striking high peak on the third chromosome in the La Lopé comparison. Most of the annotated genes in the peak region do not have an apparent connection with habitat adaptation (Additional file 1: Table S4). One gene of possible interest is a cytochrome P450 gene (AAEL003890 on VectorBase, or CYP6AG8 [60, 62]), which could be related to detoxication and insecticide resistance [63–67]. Further studies specifically focusing on this gene are needed to further confirm whether this gene was under diverging selection pressure related to habitats. Other possibilities that cause this peak of divergence include unaccounted external gene flow into the La Lopé populations, as well as possible chromosome inversions that accumulate genetic divergence [68–70]. This divergent genomic region was not found in the analysis of the Rabai populations (Fig. 5b) or the La Lopé samples from 2014 (data not shown). Therefore, it is likely specific to the La Lopé populations in 2016 instead of representing a general mechanism of adaptation to the forest or human-made habitats.

Analysis in this study using a panel of ~17,000–25,000 SNP loci and 12 microsatellite loci reached the same general conclusions, which again proves the robustness of using these genetic markers in studies of the population genetic structure of *Ae. aegypti* [32, 71]. Multiallelic loci like microsatellite and biallelic loci like SNP could behave differently due to their different nature (e.g. mutation rates) [68]. The congruence between them further supports the lack of detectable genetic differentiation between mosquitoes in the forest and anthropogenic habitats. When comparing the two types of markers, the SNP data has the advantage of allowing individual-based analysis, such as estimating kinship coefficients, which could contribute to addressing fine-scale questions. For example, in this study, we observed decreasing kinship between mosquito individuals further away from each other (Additional file 1: Figure S6a). In another recent study, Jasper et al. [72] developed a new methodology to use SNP based relatedness measures and spatial data to estimate dispersal in *Ae. aegypti*. Furthermore, the genome-wide SNP data allow explorations of variations across the genome and thus opens the opportunity to identify the genetic basis associated with any environmental changes. An intriguing demonstration is a recent study by Endersby-Harshman et al. [25], which found a genomic region strongly related to insecticide resistance. The sliding window *F*_*st*_ analysis used here also provides some hints in this regard. Similar analyses could further benefit from using whole-genomic sequencing data providing a higher resolution.

## Conclusions

The genetic similarities between *Ae. aegypti* collected in forests and villages in La Lopé and Rabai suggest that colonizing different habitats does not necessarily accompany or require substantial genomic differentiation. This is consistent with previous observations of locally domesticated *Aaf* in multiple locations in Africa [17–19]. The finding is relevant for disease monitoring and control. Although this study did not explicitly test migration, the lack of genetic differentiation and the proximity of forest and village *Ae. aegypti* suggested a possibility of gene flow between habitats. If this is the case, mosquito surveillance and control could benefit from considering the sylvatic mosquito populations. For instance, control programmes like insecticide application usually focus on mosquito populations closely associated with humans, but these alone would not be effectual if the sylvatic habitats are reservoirs of potentially domestic-living *Ae. aegypti*. Also, if mosquitoes readily move between habitats, they could potentially introduce new pathogens or strains of viruses from the forest reservoirs to the human communities, which could lead to unexpected epidemiological consequences. In addition, this absence of habitat-associated genetic barriers in Africa raises interesting questions about the evolutionary history of *Ae. aegypti*. For example, when and how did the domestic adaptations come about in the process of *Ae. aegypti* becoming a human specialist if switching habitats does not necessarily link to genomic differentiation? Why do the *Aaa* populations outside of Africa rarely move back into sylvatic habitats with only a few exceptions, such as in the Caribbean and Argentina [73, 74]? Will the village- and urban-living *Aaf* in Africa evolve to be more domesticated in parallel with their American and Asian counterparts? Addressing these questions could deepen our understanding of the global invasion of *Ae. aegypti* and their successful utilization of the domestic habitats. It could also provide insights into improving mosquito control, especially in African countries where habitat shifts occur repeatedly.

## Supplementary information

**Additional file 1: Table S1.** Information on the global panel of *Ae. aegypti* populations included in this study. **Table S2.** Allelic richness, private allelic richness, expected heterozygosity (He) and observed heterozygosity (Ho) of La Lopé and Rabai samples grouped by habitats or sampling sites. **Table S3.** Pairwise *F*_*st*_ of La Lopé and Rabai samples grouped by habitats or sampling sites. **Table S4.** Annotated genes in the region of the *F*_*st*_ peak in La Lopé populations. **Figure S1.** Geographical locations of the global panel of *Ae. aegypti* populations included in this study. **Figure S2.** Genetic ancestry of the La Lopé and Rabai populations with a reference panel of global populations of *Ae. aegypti*. **Figure S3.** PCA of the La Lopé and Rabai populations with the global panel of populations. **Figure S4.** Genetic structure of the Rabai mosquito samples grouped by sampling sites. **Figure S5.** Results of STRUCTURE analysis and PCA using microsatellite data. **Figure S6.** Individual pairwise kinship coefficients of mosquito samples from Rabai. **Figure S7.** Phylogeny of the La Lopé and Rabai mosquitoes.

## Data Availability

The microsatellite and SNP data of the new samples from Gabon and Kenya were stored on VectorBase.org with project ID VBP0000625. The microsatellite and SNP data of the reference panel can be found on VectorBase.org with project ID VBP0000138 and VBP0000295.

## Abbreviations

Aaa: *Aedes aegypti aegypti*
Aaf: *Aedes aegypti formosus*
ANOVA: Analysis of variance
MCMC: Markov chain Monte Carlo
PCA: Principal components analysis
F_st_: Fixation index
He: Expected heterozygosity
Ho: Observed heterozygosity
SNPs: Single nucleotide polymorphisms
