# Supplementary material for: Genetic structure of the mosquito Aedes aegypti in local forest and domestic habitats in Gabon and Kenya
Source: Parasit Vectors. 2020 Aug 13;13:417. doi: 10.1186/s13071-020-04278-w (PMC7427282; doi:10.1186/s13071-020-04278-w)
Supplement: Supplementary file 1 — Additional file 1: Table S1. Information on the global panel of Ae. aegypti populations included in this study. Table S2. Allelic richness, private allelic richness, expected heterozygosity (He) and observed heterozygosity (Ho) of La Lopé and Rabai samples grouped by habitats or sampling sites. Table S3. Pairwise Fst of La Lopé and Rabai samples grouped by habitats or sampling sites. Table S4. Annotated genes in the region of the Fst peak in La Lopé populations. Figure S1. Geographical locations of the global panel of Ae. aegypti populations included in this study. Figure S2. Genetic ancestry of the La Lopé and Rabai populations with a reference panel of global populations of Ae. aegypti. Figure S3. PCA of the La Lopé and Rabai populations with the global panel of populations. Figure S4. Genetic structure of the Rabai mosquito samples grouped by sampling sites. Figure S5. Results of STRUCTURE analysis and PCA using microsatellite data. Figure S6. Individual pairwise kinship coefficients of mosquito samples from Rabai. Figure S7. Phylogeny of the La Lopé and Rabai mosquitoes. [file 13071_2020_4278_MOESM1_ESM.docx]

**Additional file 1**

**Table S1.** Information on the global panel of *Ae. aegypti* populations included in this study

| **Region** | **Country/Area** | **Population** | **ID ^a^** | **Samples** | **Latitude** | **Longitude** | **Year** |
| --- | --- | --- | --- | --- | --- | --- | --- |
| Africa | Guinea-Bissau (GW) | Bijagos | 1 | 30 / 0 ^b^ | 11.250 | -16.083 | 2009 |
|  | Senegal (SN) | Ngari | 2 | 16 / 0 | 14.744 | -17.512 | 2012 |
|  |  | Dakar | 3 | 30 / 0 | 14.694 | -17.444 | 2005 |
|  |  | N'goye | 4 | 30 / 0 | 14.642 | -16.435 | 2007 |
|  |  | Sedhiou | 5 | 30 / 9 | 12.707 | -15.555 | 2012 |
|  |  | Koungheul | 6 | 30 / 0 | 13.980 | -14.802 | 2006 |
|  |  | Goudiry | 7 | 30 / 9 | 14.184 | -12.717 | 2012 |
|  |  | Kedougou | 8 | 30 / 0 | 12.561 | -12.175 | 2006 |
|  | Cameroon (CM) | Yaounde Mokolo | 9 | 0 / 5 | 3.873 | 11.501 | 2015 |
|  |  | Yaounde MvogAda | 10 | 0 / 7 | 3.863 | 11.526 | 2015 |
|  |  | Yaounde Center | 11 | 30 / 8 | 3.867 | 11.517 | 2014 |
|  |  | Yaounde Forest | 12 | 0 / 7 | 3.876 | 11.376 | 2015 |
|  |  | Yaounde Village | 13 | 0 / 8 | 3.861 | 11.394 | 2015 |
|  |  | Buffalo camp | 14 | 0 / 8 | 8.371 | 13.866 | 2014 |
|  | Gabon (GA) | La Lopé forest | 15 | 0 / 11 | -0.379 | 11.527 | 2014 |
|  |  | La Lopé village | 16 | 0 / 9 | -0.379 | 11.527 | 2014 |
|  |  | La Lopé forest  2016 | 17 | 30 / 11 | -0.176 | 11.607 | 2016 |
|  |  | La Lopé village 2016 | 18 | 30 / 8 | -0.106 | 11.607 | 2016 |
|  |  | Franceville | 19 | 30 / 12 | -1.633 | 13.583 | 2014 |
|  | Angola (AO) | Luanda | 20 | 28 / 8 | -9.767 | 14.267 | 2016 |
|  | South Africa (ZA) | Johannesburg | 21 | 18 / 7 | -25.990 | 27.901 | 2015 |
|  | Uganda (UG) | Kichwamba | 22 | 30 / 0 | -0.221 | 30.098 | 2009 |
|  |  | Bundibugyo | 23 | 30 / 0 | 0.708 | 30.064 | 2009 |
|  |  | Zika village | 24 | 0 / 10 | 0.127 | 32.531 | 2016 |
|  |  | Lunyo | 25 | 30 / 7 | 0.072 | 32.463 | 2013 |
|  | Kenya (KE) | Kakamega | 26 | 8 / 0 | 0.283 | 34.752 | 2012 |
|  |  | Kisumu | 27 | 30 / 5 | -0.092 | 34.768 | 2012 |
|  |  | Nairobi | 28 | 30 / 7 | -1.290 | 36.818 | 2012 |
|  |  | Garissa | 29 | 19 / 0 | -0.453 | 39.646 | 2012 |
|  |  | Mombasa | 30 | 30 / 0 | -4.044 | 39.668 | 2012 |
|  |  | Kaya Forest | 31 | 0 / 10 | -3.932 | 39.596 | 2012 |
|  |  | Rabai-out | 32 | 30 / 0 | -3.932 | 39.596 | 2009 |
|  |  | Rabai-in | 33 | 30 / 9 | -4.049 | 39.671 | 2012 |
|  |  | Rabai forest  2017 | 34 | 30 / 11 | -3.930 | 39.596 | 2017 |
|  |  | Rabai peridomestic 2017 | 35 | 22 / 12 | -3.929 | 39.571 | 2017 |
|  |  | Rabai domestic 2017 | 36 | 30 / 18 | -3.929 | 39.571 | 2017 |
| America | United States (USA) | Miami | 37 | 20 / 0 | 25.762 | -80.192 | 2011 |
|  | Dominica (DM) | Dominica | 38 | 20 / 9 | 15.592 | -61.411 | 2009 |
|  | Costa Rica (CR) | Siquirres | 39 | 20 / 5 | 9.938 | -84.095 | 2014 |
|  | Colombia (CO) | Cali | 40 | 30 / 7 | 3.439 | -76.516 | 2013 |
|  | Brazil (BR) | Maraba | 41 | 30 / 0 | -5.374 | -49.130 | 2010 |
|  |  | Macapà | 42 | 0 / 12 | 0.035 | -51.071 | \ ^c^ |
|  | Mexico (ME) | Chetumal lab | 43 | 0 / 8 | \ ^c^ | \ ^c^ | 2005 |
| Asia-Europe-Pacific | Georgia (GE) | Georgia | 44 | 0 / 5 | 41.961 | 43.362 | 2015 |
|  | Portugal (PO) | Madeira | 45 | 20 / 0 | 32.761 | -16.960 | 2012 |
|  | Vietnam (VT) | Ho Chi Minh City | 46 | 20 / 12 | 10.803 | 106.695 | 2013 |
|  | Philippines (PH) | Philippines | 47 | 20 / 7 | 10.283 | 123.947 | 2013 |
|  | Australia (AU) | Cairns | 48 | 0 / 7 | -16.817 | 145.686 | 2013 |
|  |  | Townsville | 49 | 20 / 0 | -19.259 | 146.817 | 2009 |
|  | French Polynesia (FP) | Tahiti | 50 | 20 / 11 | -17.531 | -149.558 | 2010 |

^a^ Population indexes

^b^ Number of samples in the microsatellite data / number of samples in the SNP data

^c^ Information is not available.

**Table S2.** Allelic richness, private allelic richness, expected heterozygosity (*He*) and observed heterozygosity (*Ho*) of La Lopé and Rabai samples grouped by habitats or sampling sites.

| **Group** | **Allelic richness ^a^** | **Private alleles ^a^** | **He (microsatellite)** | **Ho (microsatellite)** | **He (SNP)** | **Ho (SNP)** |
| --- | --- | --- | --- | --- | --- | --- |
| La Lopé forest | 6.73 | 1.57 | 0.693 | 0.622 | 0.273 | 0.245 |
| La Lopé village | 5.87 | 0.71 | 0.651 | 0.644 | 0.264 | 0.246 |
| Rabai forest | 7.76 | 0.99 | 0.732 | 0.656 | 0.229 | 0.203 |
| Rabai peridomestic | 8 | 0.95 | 0.697 | 0.644 | 0.239 | 0.208 |
| Rabai domestic | 7.79 | 1.29 | 0.704 | 0.608 | 0.244 | 0.216 |
| Kaya Bomu forest |  |  |  |  | 0.229 | 0.203 |
| Chang’ombe |  |  |  |  | 0.236 | 0.212 |
| Mbarekani |  |  |  |  | 0.240 | 0.214 |
| Bengo |  |  |  |  | 0.242 | 0.209 |
| Kwa Bendegwa |  |  |  |  | 0.242 | 0.217 |

^a^ Average values of the 12 microsatellite loci, estimated in HPRARE with rarefaction to the minimum sample size (44 genes)

**Table S3.** Pairwise *F_st_* of La Lopé and Rabai samples grouped by habitats or sampling sites.

| **Group 1** | **Group 2** | ***F_st_* (microsatellite)** | ***F_st_* (SNP)** |
| --- | --- | --- | --- |
| La Lopé forest | La Lopé village | 0.0336^*^ | 0.0086 |
| Rabai forest | Rabai peridomestic | 0.0068 | 0.0042 |
| Rabai forest | Rabai domestic | 0.0066^*^ | 0.0079 |
| Rabai peridomestic | Rabai domestic | 0.0100^*^ | -0.0004 |
| Kaya Bomu Forest | Chang’ombe |  | 0.0031 |
| Kaya Bomu Forest | Mbarekani |  | 0.0058 |
| Kaya Bomu Forest | Bengo |  | 0.0114 |
| Kaya Bomu Forest | Kwa Bendegwa |  | 0.0198 |
| Chang’ombe | Mbarekani |  | 0.0002 |
| Chang’ombe | Bengo |  | 0.0038 |
| Chang’ombe | Kwa Bendegwa |  | 0.0119 |
| Mbarekani | Bengo |  | -0.0031 |
| Mbarekani | Kwa Bendegwa |  | 0.0095 |
| Bengo | Kwa Bendegwa |  | 0.0076 |

^*^ *F_st_* values that are statistically significant determined by *genepop*.

**Table S4.** Annotated genes in the region of the *Fst* peak in La Lopé populations

| **Gene ID** | **Gene name** | **Gene description** | **Gene start (bp)** | **Gene**  **end (bp)** |
| --- | --- | --- | --- | --- |
| [AAEL003867](https://www.vectorbase.org/aedes_aegypti_lvpagwg/Gene/Summary?db=core;g=AAEL003867) |  |  | [84985916](https://www.vectorbase.org/aedes_aegypti_lvpagwg/contigview?chr=3&vc_start=84985916&vc_end=84998389) | [84998389](https://www.vectorbase.org/aedes_aegypti_lvpagwg/contigview?chr=3&vc_start=84985916&vc_end=84998389) |
| [AAEL003891](https://www.vectorbase.org/aedes_aegypti_lvpagwg/Gene/Summary?db=core;g=AAEL003891) |  | Ctl transporter | [85012958](https://www.vectorbase.org/aedes_aegypti_lvpagwg/contigview?chr=3&vc_start=85012958&vc_end=85036685) | [85036685](https://www.vectorbase.org/aedes_aegypti_lvpagwg/contigview?chr=3&vc_start=85012958&vc_end=85036685) |
| [AAEL003901](https://www.vectorbase.org/aedes_aegypti_lvpagwg/Gene/Summary?db=core;g=AAEL003901) |  |  | [85043660](https://www.vectorbase.org/aedes_aegypti_lvpagwg/contigview?chr=3&vc_start=85043660&vc_end=85088513) | [85088513](https://www.vectorbase.org/aedes_aegypti_lvpagwg/contigview?chr=3&vc_start=85043660&vc_end=85088513) |
| [AAEL003887](https://www.vectorbase.org/aedes_aegypti_lvpagwg/Gene/Summary?db=core;g=AAEL003887) |  | Vacuolar membrane protein pep11 | [85091046](https://www.vectorbase.org/aedes_aegypti_lvpagwg/contigview?chr=3&vc_start=85091046&vc_end=85100508) | [85100508](https://www.vectorbase.org/aedes_aegypti_lvpagwg/contigview?chr=3&vc_start=85091046&vc_end=85100508) |
| [AAEL003874](https://www.vectorbase.org/aedes_aegypti_lvpagwg/Gene/Summary?db=core;g=AAEL003874) |  |  | [85116735](https://www.vectorbase.org/aedes_aegypti_lvpagwg/contigview?chr=3&vc_start=85116735&vc_end=85135667) | [85135667](https://www.vectorbase.org/aedes_aegypti_lvpagwg/contigview?chr=3&vc_start=85116735&vc_end=85135667) |
| [AAEL003872](https://www.vectorbase.org/aedes_aegypti_lvpagwg/Gene/Summary?db=core;g=AAEL003872) | [Tctp](https://www.vectorbase.org/aedes_aegypti_lvpagwg/Gene/Summary?db=core;g=AAEL003872) | Translationally controlled tumor protein homolog (TCTP) | [85147401](https://www.vectorbase.org/aedes_aegypti_lvpagwg/contigview?chr=3&vc_start=85147401&vc_end=85160385) | [85160385](https://www.vectorbase.org/aedes_aegypti_lvpagwg/contigview?chr=3&vc_start=85147401&vc_end=85160385) |
| [AAEL003873](https://www.vectorbase.org/aedes_aegypti_lvpagwg/Gene/Summary?db=core;g=AAEL003873) |  | Glycerol-3-phosphate dehydrogenase | [85191823](https://www.vectorbase.org/aedes_aegypti_lvpagwg/contigview?chr=3&vc_start=85191823&vc_end=85266305) | [85266305](https://www.vectorbase.org/aedes_aegypti_lvpagwg/contigview?chr=3&vc_start=85191823&vc_end=85266305) |
| [AAEL003875](https://www.vectorbase.org/aedes_aegypti_lvpagwg/Gene/Summary?db=core;g=AAEL003875) |  | transcription factor SP4, putative | [85282197](https://www.vectorbase.org/aedes_aegypti_lvpagwg/contigview?chr=3&vc_start=85282197&vc_end=85302730) | [85302730](https://www.vectorbase.org/aedes_aegypti_lvpagwg/contigview?chr=3&vc_start=85282197&vc_end=85302730) |
| [AAEL025841](https://www.vectorbase.org/aedes_aegypti_lvpagwg/Gene/Summary?db=core;g=AAEL025841) |  |  | [85323409](https://www.vectorbase.org/aedes_aegypti_lvpagwg/contigview?chr=3&vc_start=85323409&vc_end=85324281) | [85324281](https://www.vectorbase.org/aedes_aegypti_lvpagwg/contigview?chr=3&vc_start=85323409&vc_end=85324281) |
| [AAEL003899](https://www.vectorbase.org/aedes_aegypti_lvpagwg/Gene/Summary?db=core;g=AAEL003899) |  | Sugar transporter | [85352740](https://www.vectorbase.org/aedes_aegypti_lvpagwg/contigview?chr=3&vc_start=85352740&vc_end=85370365) | [85370365](https://www.vectorbase.org/aedes_aegypti_lvpagwg/contigview?chr=3&vc_start=85352740&vc_end=85370365) |
| [AAEL003894](https://www.vectorbase.org/aedes_aegypti_lvpagwg/Gene/Summary?db=core;g=AAEL003894) | [GNBPB5](https://www.vectorbase.org/aedes_aegypti_lvpagwg/Gene/Summary?db=core;g=AAEL003894) | Gram-Negative Binding Protein (GNBP) or Beta-1 3-Glucan Binding Protein (BGBP). | [85381249](https://www.vectorbase.org/aedes_aegypti_lvpagwg/contigview?chr=3&vc_start=85381249&vc_end=85389978) | [85389978](https://www.vectorbase.org/aedes_aegypti_lvpagwg/contigview?chr=3&vc_start=85381249&vc_end=85389978) |
| [AAEL003889](https://www.vectorbase.org/aedes_aegypti_lvpagwg/Gene/Summary?db=core;g=AAEL003889) | [GNBPB1](https://www.vectorbase.org/aedes_aegypti_lvpagwg/Gene/Summary?db=core;g=AAEL003889) | Gram-Negative Binding Protein (GNBP) or Beta-1 3-Glucan Binding Protein (BGBP). | [85412482](https://www.vectorbase.org/aedes_aegypti_lvpagwg/contigview?chr=3&vc_start=85412482&vc_end=85421308) | [85421308](https://www.vectorbase.org/aedes_aegypti_lvpagwg/contigview?chr=3&vc_start=85412482&vc_end=85421308) |
| [AAEL003890](https://www.vectorbase.org/aedes_aegypti_lvpagwg/Gene/Summary?db=core;g=AAEL003890) |  | Cytochrome P450 | [85683648](https://www.vectorbase.org/aedes_aegypti_lvpagwg/contigview?chr=3&vc_start=85683648&vc_end=85685698) | [85685698](https://www.vectorbase.org/aedes_aegypti_lvpagwg/contigview?chr=3&vc_start=85683648&vc_end=85685698) |
| [AAEL019766](https://www.vectorbase.org/aedes_aegypti_lvpagwg/Gene/Summary?db=core;g=AAEL019766) |  |  | [86037281](https://www.vectorbase.org/aedes_aegypti_lvpagwg/contigview?chr=3&vc_start=86037281&vc_end=86791700) | [86791700](https://www.vectorbase.org/aedes_aegypti_lvpagwg/contigview?chr=3&vc_start=86037281&vc_end=86791700) |
| [AAEL014372](https://www.vectorbase.org/aedes_aegypti_lvpagwg/Gene/Summary?db=core;g=AAEL014372) |  | Juvenile hormone-inducible protein, putative | [86733087](https://www.vectorbase.org/aedes_aegypti_lvpagwg/contigview?chr=3&vc_start=86733087&vc_end=86734639) | [86734639](https://www.vectorbase.org/aedes_aegypti_lvpagwg/contigview?chr=3&vc_start=86733087&vc_end=86734639) |
| [AAEL021562](https://www.vectorbase.org/aedes_aegypti_lvpagwg/Gene/Summary?db=core;g=AAEL021562) |  |  | [86931875](https://www.vectorbase.org/aedes_aegypti_lvpagwg/contigview?chr=3&vc_start=86931875&vc_end=87006061) | [87006061](https://www.vectorbase.org/aedes_aegypti_lvpagwg/contigview?chr=3&vc_start=86931875&vc_end=87006061) |

Source: vectorbase.org


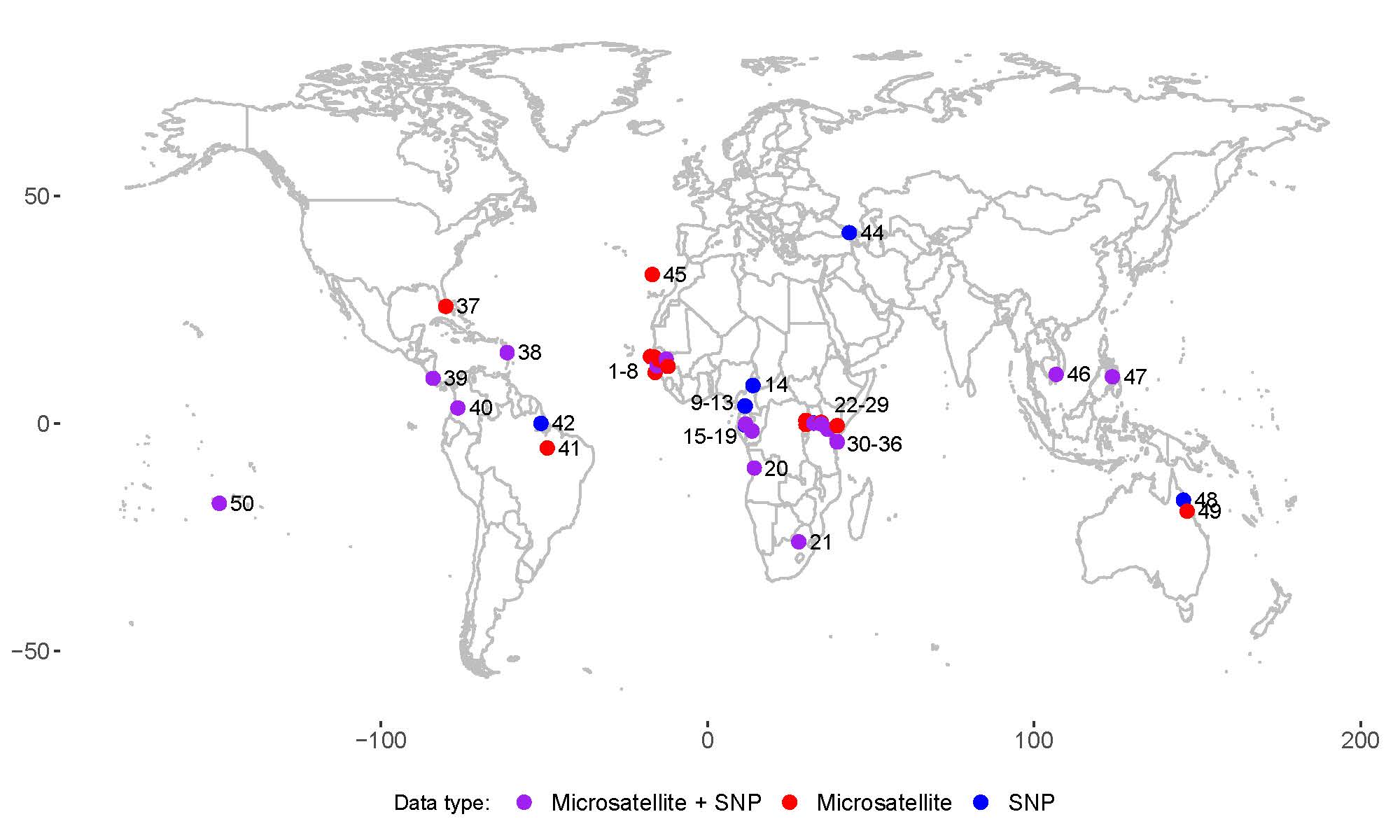


**Figure S1.** Geographic locations of the global panel of *Ae. aegypti* populations included in this study. The number that labels each population corresponds to the population index in Table S1 (column “ID”). The color of each point represents the availability of microsatellite and SNP data for that population.


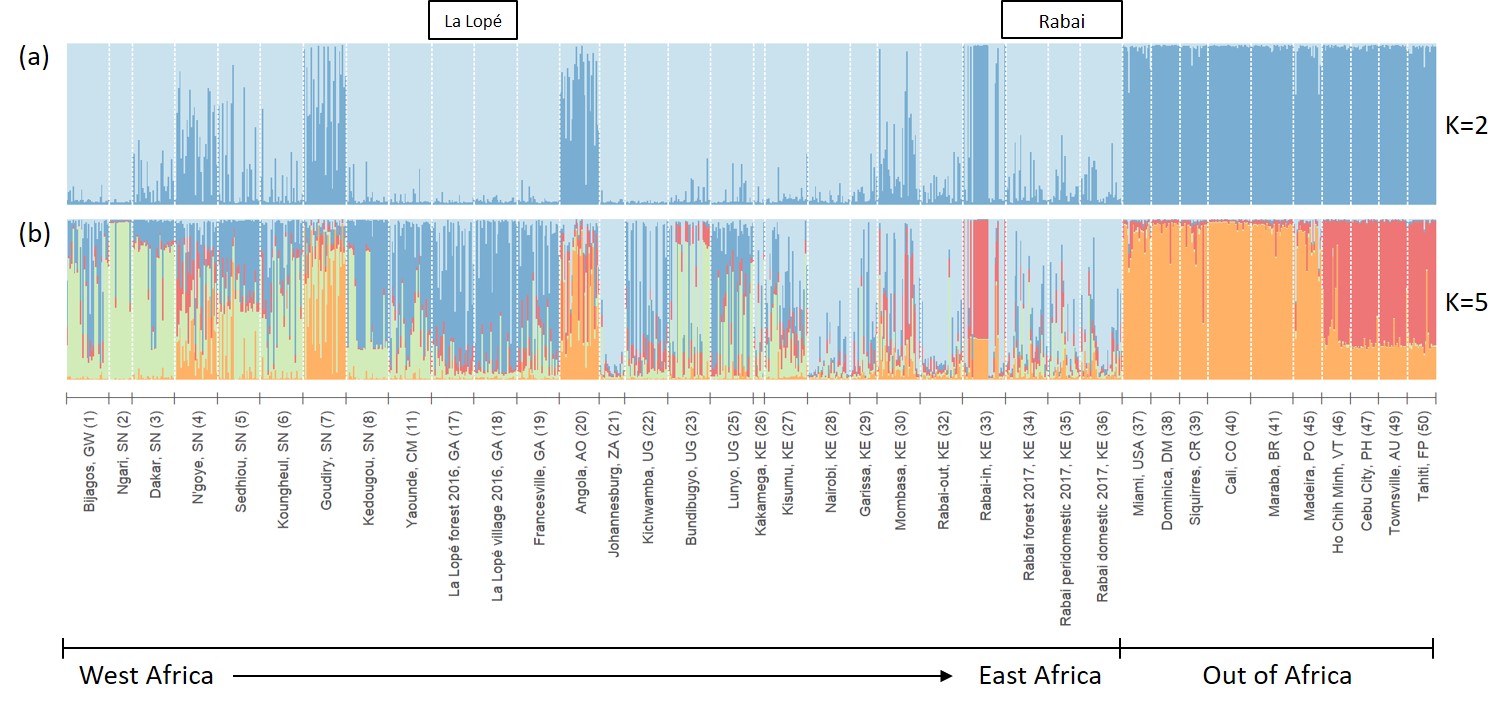


**Figure S2.** Genetic ancestry of the La Lopé and Rabai populations with a reference panel of global populations of *Ae. aegypti*, estimated using the 12 microsatellite loci in STRUCTURE with K=2 (**a**) and K=5 (**b**). Each bar represents one individual. Different colors represent different genetic clusters, and the proportion of a color indicates the probability of an individual assigned to that genetic cluster. The reference panel was selected from Gloria-Soria et al. 2016. Population names with country abbreviations and regions are listed on the x-axis. The numbers in the parentheses correspond to the population index in Table S1 (column “ID”) as well as the numbers in Figure S1. La Lopé and Rabai samples collected in 2016 and 2017 are indicated by the box on top of the figure.


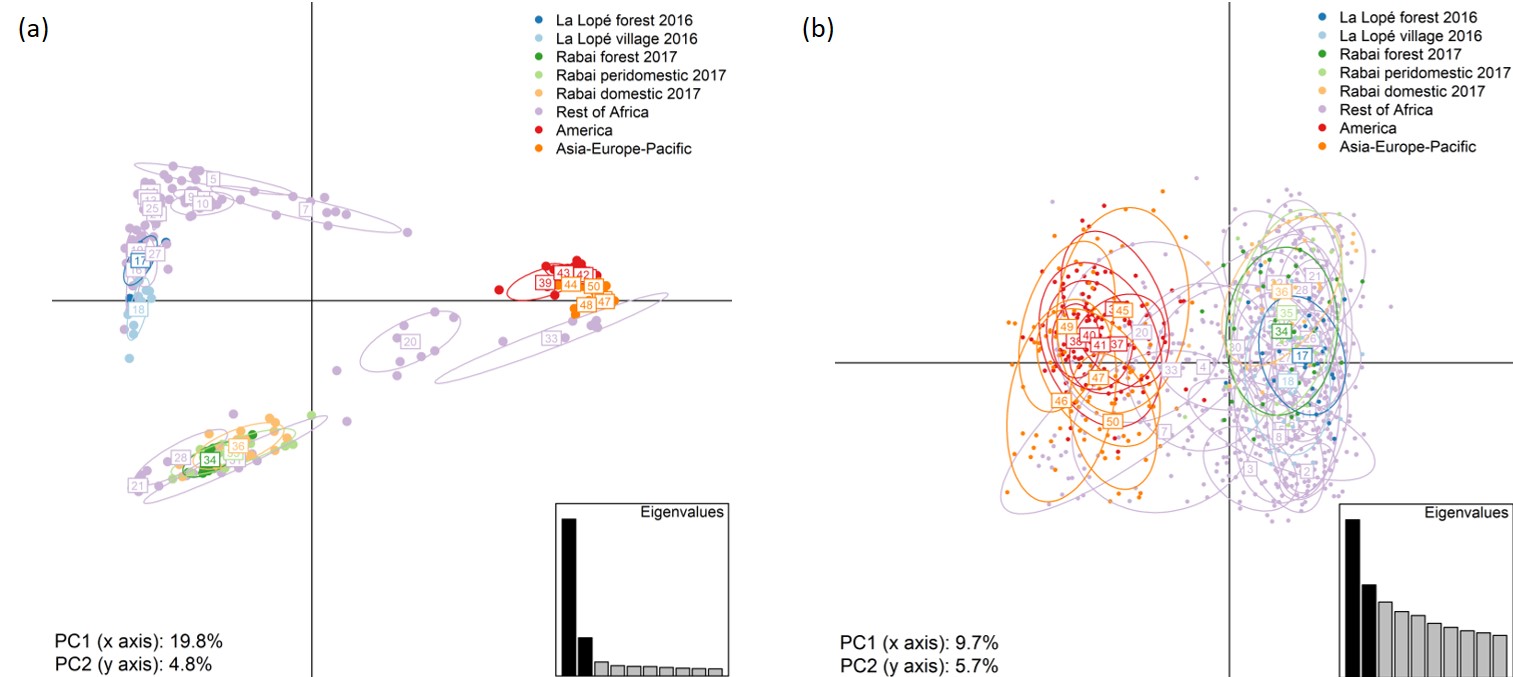


**Figure S3.** PCA of the La Lopé and Rabai populations with the global panel of populations using (**a**) 23,767 SNPs and (**b**) 12 microsatellite loci. The first two principal components (PCs) were shown with the percentage of total genetic variation explained by them labeled at the bottom left of each panel. The color of the points represents the regions of samples (Table S1) with the La Lopé 2016 and Rabai 2017 collections labeled by different colors. Eclipses were drawn for each population with a 67% probability. Populations were labeled by their indexes (Table S1, column ID), which also match the numbers in Figure S1. The inset at the bottom right of each panel shows the eigenvalues of the top 10 PCs with the first two PCs marked in black. The PCA was performed in the R package *adegenet.*


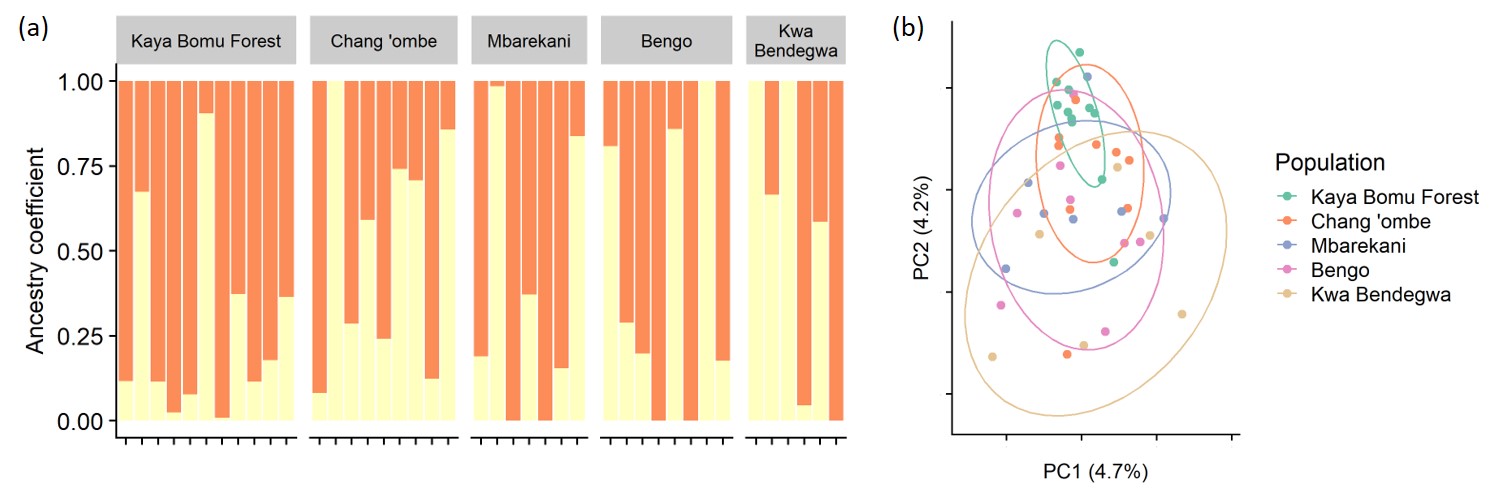


**Figure S4.** Genetic structure of the Rabai mosquito samples grouped by sampling sites generated using 23,068 SNP loci. (**a**) Results of the LEA analysis with K=2. The sampling sites are labeled on top of each bar plot. (**b**) PCA biplots by *LEA* showing the first two principal components (PCs). The numbers in the parentheses on the axes indicate the percentage of total variation explained by the PCs. Eclipses were drawn with an 80% confidence level.


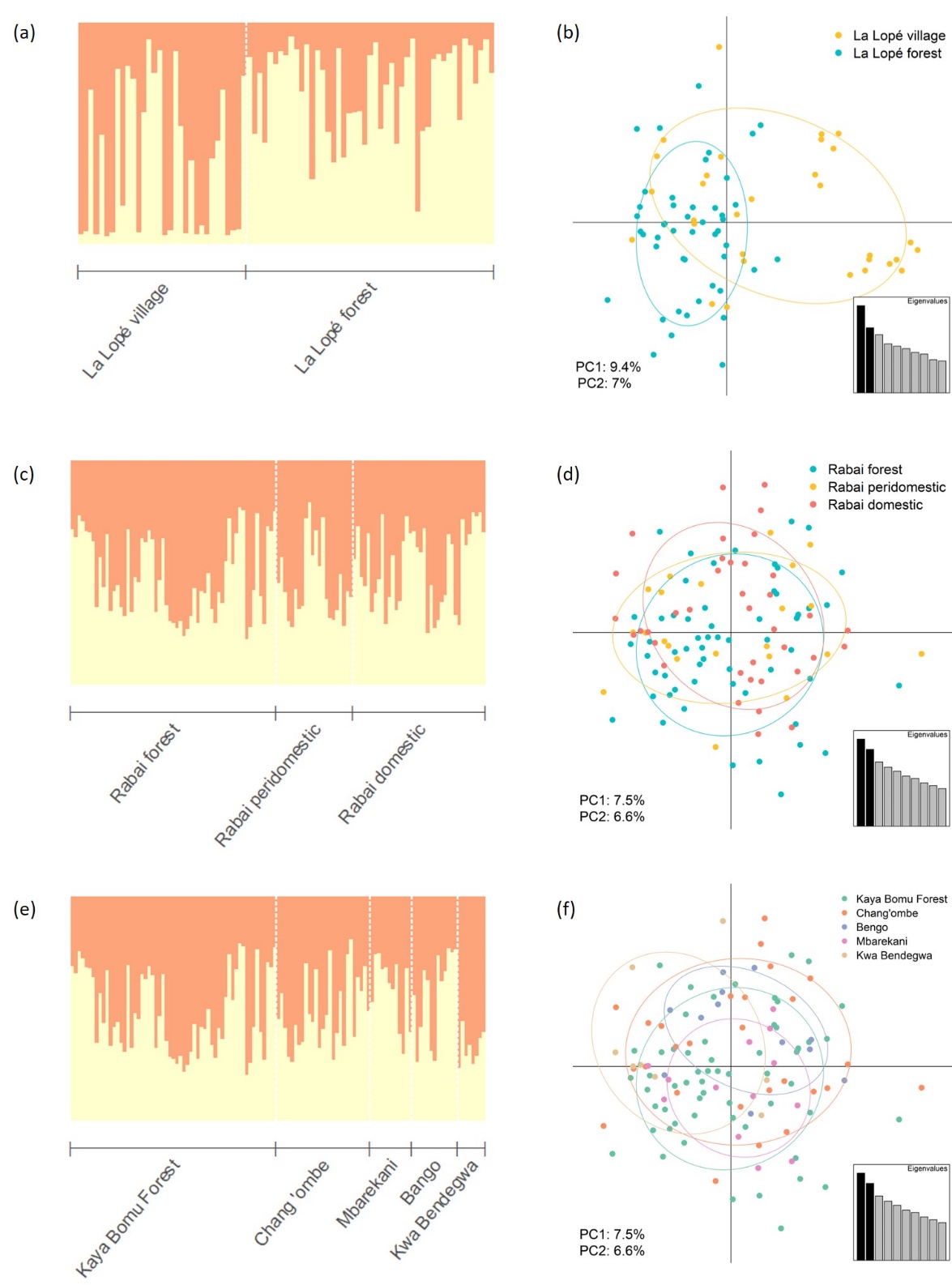


**Figure S5.** Genetic structure of the La Lopé (**a**, **b**) and Rabai (**c**-**f**) mosquitoes grouped by sampling sites or habitats, generated using the 12 microsatellite loci. (**a**, **c**, **e**) Results of STRUCTURE analysis with K=2. The groups are labeled on the bottom of each bar plot. (**b**, **d**, **f**) PCA biplots by *adegenet* showing the first two principal components (PCs). Eclipses were drawn with a 67% probability of inclusion. The inset in each biplot shows the eigenvalues of the top 10 PCs with the first two PCs marked in black. The percentage of total variance explained by each of the two PCs is labeled at the bottom left.


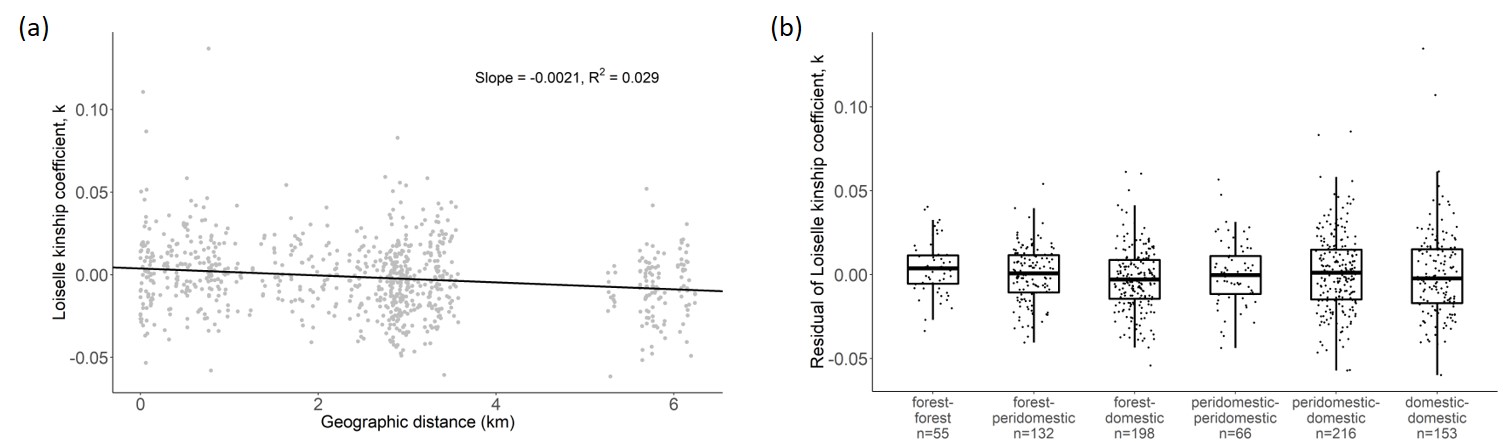


**Figure S6.** Individual pairwise kinship coefficients of mosquito samples from Rabai. Each point represents one mosquito pair. (**a**) Correlation between geographic distance and kinship coefficients. The linear regression is used to remove this distance effect is represented by the line, with the slope and *R^2^* values labeled. (**b**) Residuals of the kinship coefficients in Rabai after removing distance effects categorized by habitats, with the number of pairs labeled on the x-axis. The boxplots show the median (the horizontal bar), interquartile range (IQR, the box), and 1.5 x IQR above and below the IQR (the vertical bar). ANOVA did not find significant difference between categories (F = 1.337, bootstrap p = 0.211).


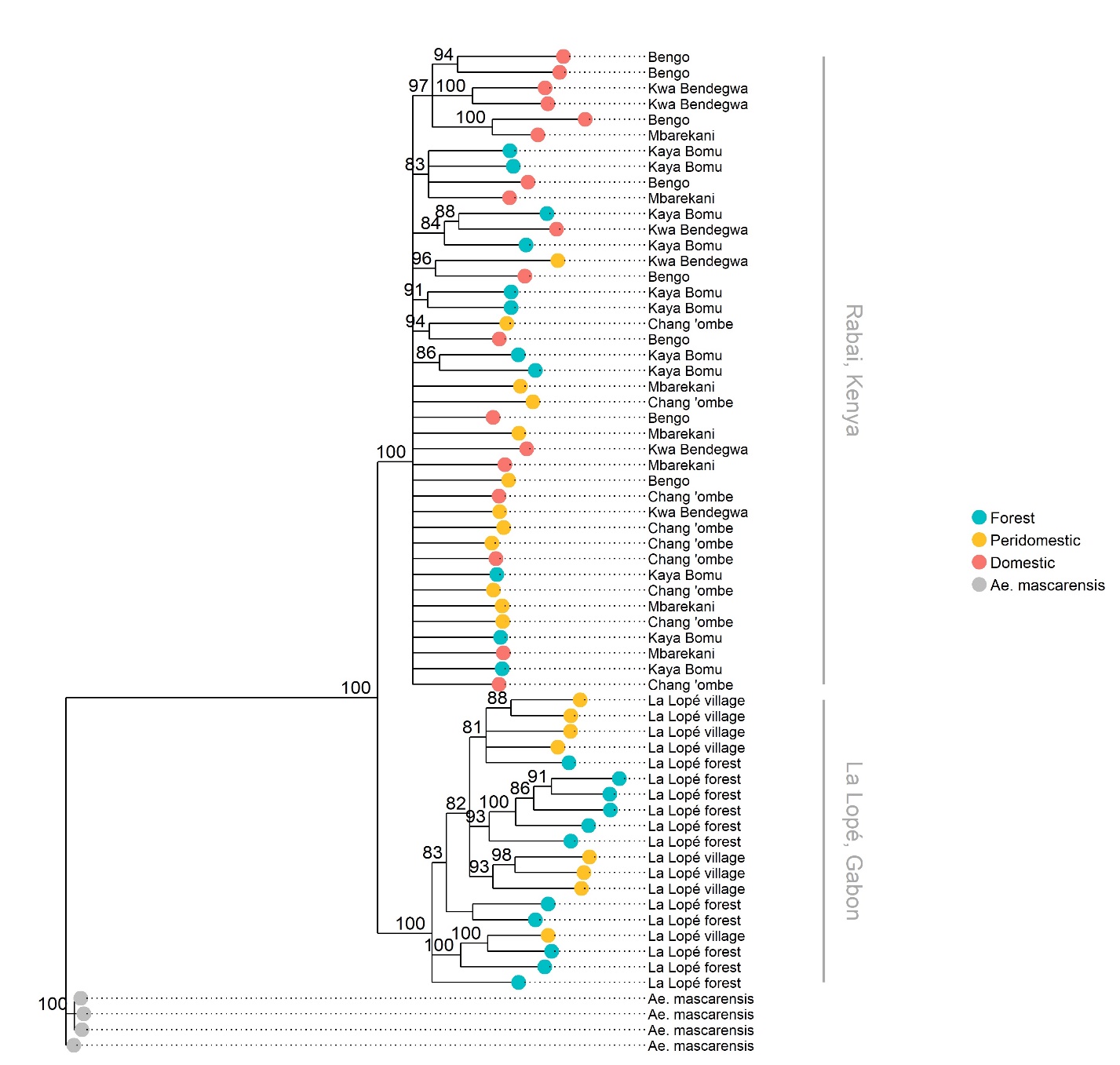


**Figure S7.** Phylogeny of the La Lopé and Rabai mosquitoes generated in IQTree with 22,287 SNP loci. Four *Ae. mascarensis* samples were used as the outgroup. The number on each node indicates the support level estimated by 1000 ultrafast bootstraps and nodes with support values smaller than 80 are collapsed. The colors of the tips represent the habitats, and the labels indicate the sampling sites.
